# Paroxysmal Atrial Fibrillation Originating From the Inferior Vena Cava: A Case Report and Literature Review

**DOI:** 10.3389/fcvm.2022.935524

**Published:** 2022-07-04

**Authors:** Yirao Tao, Donghui Yang, Liang Chen

**Affiliations:** Department of Cardiology, The Second Affiliated Hospital of Dalian Medical University, Dalian, China

**Keywords:** atrial fibrillation, inferior vena cava, trigger, driver, catheter ablation

## Abstract

Atrial fibrillation is a common arrhythmia, but atrial fibrillation originating in the inferior vena cava is extremely rare. Here, we present a case of a 51-year-old woman with symptomatic paroxysmal atrial fibrillation, who was admitted to the Second Affiliated Hospital of Dalian Medical University and underwent radiofrequency ablation. The atrial fibrillation persisted despite pulmonary vein isolation. The inferior vena cava was then identified not only as a trigger but also as the driver to maintain atrial fibrillation, and tachycardia terminated successfully by discharging at the inferior vena cava. Furthermore, we performed a literature review of five previous case reports on this subject.

## Highlights

- It is extremely rare for atrial fibrillation to originate from inferior vena cava. We present the first report to show inferior vena cava acts as both the trigger and the driver of atrial fibrillation using a three-dimensional imaging system.
- The electrocardiogram pattern of atrial tachycardia or premature atrial complex in individuals with paroxysmal atrial fibrillation originating from the inferior vena cava shows a narrow positive ectopic P wave in lead V1 and a negative ectopic P wave in inferior leads.
- Tachycardia terminates successfully by discharging at the inferior vena cava.

## Introduction

Paroxysmal atrial fibrillation (AF) is mostly initiated by triggers (1), usually from pulmonary veins. Therefore, pulmonary veins disconnection is the cornerstone for paroxysmal AF ablation. Nevertheless, about 11% of cases of AF are elicited by other triggers (2). These triggers include the crista terminalis, the superior vena cava, the posterior wall of the left atrium, and the coronary sinus (CS) (2). Moreover, the Marshall buddle (3) and left atrial appendage (4) have also been implicated in AF initiation. The reports about AF raising from the inferior vena cava (IVC) are extremely rare.

We presented a case of paroxysmal AF originating in the IVC, in whom the tachycardia was successfully eliminated by discharging at IVC.

## Case Presentation

**FIGURE 1:**
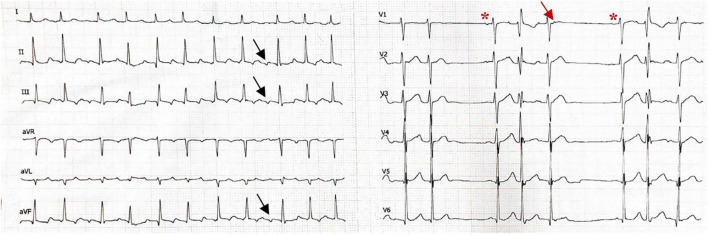
Electrocardiogram showing atrial flutter with atrial rate 280–290/min with variable atrioventricular conduction in the limb leads, the atrial wave displayed negative wave in inferior leads (II, III, and avF, black arrow). The precordial 6 lead rhythm strip shows termination of atrial flutter with a sinus beat (red asterisk), followed by four consecutive ectopic atrial beats at rate 250/min, which showed a narrow positive wave in lead V1 (red arrow). The first conducted beat occurred with the right brunch block, the second was blocked, the third conducted a with a narrow QRS, and the fourth was blocked. This followed by another sinus beat followed by the same atrial tachycardia/atrial flutter at a rate of 260/min. The ECG was recorded at a speed of 25 mm/s (x axis) and an amplitude of 10 mm/mV (y axis).

A 51-year-old woman suffered from symptomatic paroxysmal AF with a 4-month history. Her AF episodes did not respond to treatment with propafenone, and then she was admitted to the Second Affiliated Hospital of Dalian Medical University. The baseline characteristics of this patient were presented in Table 1. Her admission electrocardiogram (ECG), echocardiography, physical examination, and blood test results were unremarkable. The surface ECG recorded when the patient experienced palpitations during hospitalization presented episodes of atrial tachycardia/atrial flutter with a negative atrial wave morphology in inferior leads (II, III, and avF), and narrow positive atrial wave morphology in lead V1 (Figure 1). The patient was diagnosed with paroxysmal AF, atrial flutter, and atrial tachycardia. The diagnostic assessment is shown in Supplementary Table 1. The patient participated in a discussion and was fully informed of the state of the illness and the possible benefits and risks of various treatments and then chose radiofrequency ablation.

**TABLE 1:** Baseline characteristics.

Baseline characteristics	
Sex	Female
Age, years	51
Weight, Kg	45
Height, cm	150
BMI, kg/m^2^	20
Ethnicity	Asian
Hypertension	No
Diabetes	No
Coronary artery disease	No
Heart failure	No
Family history	No
Smoking	No
Alcohol	No
Atrial fibrillation duration, month	4
CHA_2_DS_2_-VASc score	1
HAS-BLED score	0
Antiarrhythmic drug	Propafenone
BNP, pg/mL	34.7
eGFR, mL/min. 1.73 m^2^	>90

*BMI, body mass index; BNP, B-type natriuretic peptide; eGFR, estimate glomerular filtration rate.*

**FIGURE 2:**
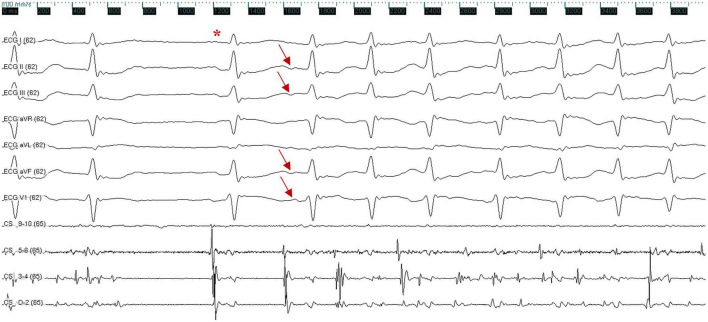
Surface and intracardiac electrocardiogram indicate the termination of atrial fibrillation, followed by a sinus beat (red asterisk); atrial fibrillation was triggered again by a premature atrial complex, which exhibited a negative P wave in inferior leads (II, III, and aVF) and a positive P wave in lead V1 (red arrow). The ECG was recorded at a speed of 100 mm/s. X axis, time line; y axis: gain. CS, coronary sinus; ECG, electrocardiogram.

**FIGURE 3:**
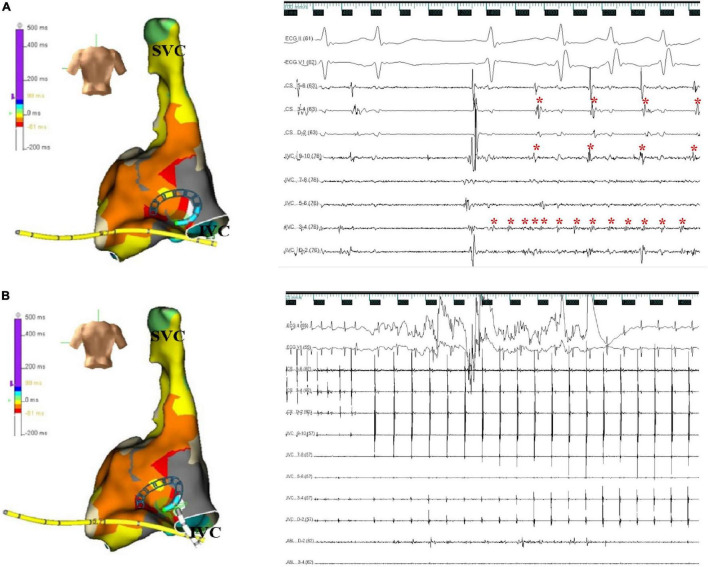
**(A)** Activation map and electrograms. Activation mapping shows that the earliest activity was at the posteromedial wall of the inferior vena cava. The intracardiac electrocardiogram indicates rapid discharges from the inferior vena cava; some of them were conducted to the right atrium (IVC 9-10) and left atrium (CS). The red asterisk represents the electrical activation recorded by the electrode. The ECG was recorded at a speed of 100 mm/s. **(B)** Successful ablation site. Atrial fibrillation terminated immediately after radiofrequency ablation at the earliest activation site in the posteromedial inferior vena cava. The ECG was recorded at a speed of 25 mm/s. X axis, time line; y axis, gain. CS, coronary sinus; ECG, electrocardiogram; IVC, inferior vena cava; SVC, superior vena cava.

The patient presented the sinus rhythm at the beginning of the radiofrequency ablation procedure. However, an AF episode started during the procedure of circumferential pulmonary veins isolation, and AF persisted after all four pulmonary veins were isolated. Tachycardia was able to terminate spontaneously, followed by several sinus beats; then, AF was triggered again by a premature atrial complex, which displayed a similar ECG appearance to atrial arrhythmia during palpitations recorded before the procedure—negative P wave in inferior leads and positive narrow P wave in lead V1 (Figure 2). Meanwhile, the CS catheter displayed a proximal-to-distal activation pattern, and the activation of the premature atrial complex on the CS catheter was not earlier than the P wave on surface ECG. Subsequently, the activation mapping procedure of the premature atrial complex in the right atrium was performed using a circular diagnostic catheter guided by the EnSite NavX system (St. Jude Medical, St. Paul, MN, United States). The superior vena cava showed passive conduction. The activation at the posteromedial wall of IVC was identified as the earliest activation, where rapid and disorganized electrical activities were recorded. Some of the electrical activities were conducted to the right atrium (IVC 9–10) and left atrium (CS) and drove tachyarrhythmia (Figure 3A). AF terminated immediately after radiofrequency ablation was performed at the earliest activation site, with a target temperature of 43^°^C, a maximal power of 40 W, and a flow rate of 20 mL/min (Figure 3B). Adenosine triphosphate infusion did not induce any atrial arrhythmia.

The patient has remained asymptomatic following the session without any need for antiarrhythmic drugs and no ECG evidence of tachycardia during the 2-month follow-up.

## Discussion

It has been established that pulmonary veins are the most common site for initiation of AF. Herein, we presented a case of paroxysmal AF originating from a very unusual location, such as the posteromedial wall of IVC. The earliest activation of the premature atrial complex which induced AF was located at the posteromedial wall of IVC by three-dimensional mapping systems, suggesting that the IVC triggered of AF. The electrical activity recorded in IVC during AF was significantly faster than in the atrium, and focal ablation of earliest atrial activation in IVC immediately terminated AF, suggesting that the IVC was also responsible for the maintenance of AF.

To the best of our knowledge, five previous reports have described AF originating from the IVC, and their baseline characteristics are summarized in Table 2 (5–9). These five case reports included six patients, aged from 22 to 83 years, and the follow-up time was from 2 to 14 months. All of the patients were free of AF during the follow-up. The majority of the cases underwent first-time AF ablation, except for a recent report (9) of a recurrent AF despite two previous AF ablations. The role of the IVC differed among these reports: it acted as the trigger in three reports and both as the trigger and the driver in two reports. Four of the five reports were published before 2010, and manipulation was mainly performed under fluoroscopy guidance. Because of the limited accuracy of fluoroscopy guidance, it is challenging to distinguish the IVC from adjacent origin locations, such as the CS ostium. Until 2021, Alonso-Martín et al. reported a case of AF originating from the IVC under three-dimensional electroanatomic mapping systems navigation (9).

**TABLE 2:** Baseline characteristics of previous case reports.

References	Year	Age	Sex	Target location of IVC	Surface ECG feature of ectopic P wave	PVI	Imaging	IVC role	IVC ablation schemes	Follow-up
Mansour et al. (5)	2002	22/60	Male/female	Posterolateral ostium	NA	No	3-D	Trigger	Foci ablation	2 months/9 months
Scavée et al. (6)	2003	44	NA	Posteromedial wall	Negative P wave in leads II, III, avF, and V1	Yes	Fluoroscopy	Trigger	IVC disconnection	14 months
Yamane et al. (7)	2005	57	Male	Anteromedial ostium	Negative P wave in leads II and III, and positive P wave in lead V1	No	Fluoroscopy	Trigger and driver	Foci ablation and IVC disconnection	12 months
Mizobuchi et al. (8)	2006	83	Female	Posterior wall	NA	No	Fluoroscopy	Trigger and driver	Foci ablation	12 months
Alonso-Martín et al. (9)	2021	47	Male	Posteromedial aspect	Negative P wave in inferior leads and positive P wave in lead V1	Yes	3-D	Trigger	Foci ablation and IVC disconnection	6 months

*3-D, 3-dimensional; ECG, electrocardiogram; IVC, inferior vena cava; NA, not available; PVI, pulmonary vein isolation.*

In a histological study from two cadavers in 1995, Hashizume et al. (10) reported that the IVC included both smooth and cardiac muscle fibers covering a length of 18 mm from the right atrium. They demonstrated that the cardiac muscle fibers in the IVC were more plentiful in the anterior aspect than in the posterior aspect. Interestingly, in our case and most of the previous cases (5, 6, 8, 9) in which AF originated in the IVC, the posterior wall was identified as the trigger. It is unknown whether this is just an accidental phenomenon or there are differences in electrical activity properties between the anterior and posterior IVC sleeves. Further investigations are required to clarify this issue.

The ECG features of ectopic P wave from the IVC were clearly visible in three of the above five reports. The ECG from our case presented a negative ectopic P wave in inferior leads, which is in line with these three cases (6, 7, 9), and the complete or dominant positive ectopic P wave in lead V1 was consistent with two of the cases (6, 9). Furthermore, all available ECG recordings displayed a narrow ectopic P wave. Several studies have demonstrated a narrow positive P wave in lead V1, suggesting that the trigger originates from the right pulmonary vein (11, 12). However, deeper inversion of P wave in inferior leads was rare even the trigger originates from the right inferior pulmonary vein. Considering that the anatomical location of the IVC is nearby the right inferior pulmonary vein but lower, we suggest that the ECG features described above may indicate the ectopic P wave originating from the IVC.

The IVC ablation schemes were not consistent among the past five case reports. In two cases (5, 8), focal ablation (like in our case) was chosen; in one case (6), IVC isolation was selected; and in two cases (7, 9), radiofrequency was applied in addition to target ablation, consequently achieving IVC isolation. All of the above patients were free of AF during the follow-up without complications. Because of the difference in anatomy and adjacent structures, the ablation strategies between non-pulmonary vein and pulmonary vein foci are different (13). The optimum ablation scheme of AF originating from the IVC remains unknown because of the limited experience, and it requires further studies.

To the best of our knowledge, this is the first report to show that IVC acts as both the trigger and the driver of AF using a three-dimensional imaging system. One limitation in the present study was that no intracardiac echocardiography or high-density mapping catheters were used. These tools may provide a more accurate assessment of the anatomical structure and more precise activation conduction mapping.

## Conclusion

We presented an extremely rare case of paroxysmal AF originating in the IVC. Moreover, we performed a literature review of five previous case reports on this subject. AF originating in the IVC is mostly initiated at the posterior wall of the IVC, and the role of the IVC is not only that of the trigger but also of the driver. The ECG pattern of AT or premature atrial complex in individuals with paroxysmal AF originating from the IVC shows a narrow positive ectopic P wave in lead V1 and a negative ectopic P wave in inferior leads. Tachycardia terminates successfully by discharging at the IVC.

## Data Availability Statement

The original contributions presented in this study are included in the article/Supplementary Material, further inquiries can be directed to the corresponding author/s.

## Ethics Statement

Written informed consent was obtained from the participant for the publication of this case report. Written informed consent was obtained from the individual(s) for the publication of any potentially identifiable images or data included in this article.

## Author Contributions

YT, LC, and DY conceived the study. YT drafted the manuscript. LC and DY checked it and performed critical revision. All authors contributed to the article and approved the submitted version.

## Conflict of Interest

The authors declare that the research was conducted in the absence of any commercial or financial relationships that could be construed as a potential conflict of interest.

## Publisher’s Note

All claims expressed in this article are solely those of the authors and do not necessarily represent those of their affiliated organizations, or those of the publisher, the editors and the reviewers. Any product that may be evaluated in this article, or claim that may be made by its manufacturer, is not guaranteed or endorsed by the publisher.
