# Supplementary figures and images for: Paroxysmal Atrial Fibrillation Originating From the Inferior Vena Cava: A Case Report and Literature Review
Source: Front Cardiovasc Med. 2022 Jul 4;9:935524. doi: 10.3389/fcvm.2022.935524 (PMC9289394; doi:10.3389/fcvm.2022.935524)

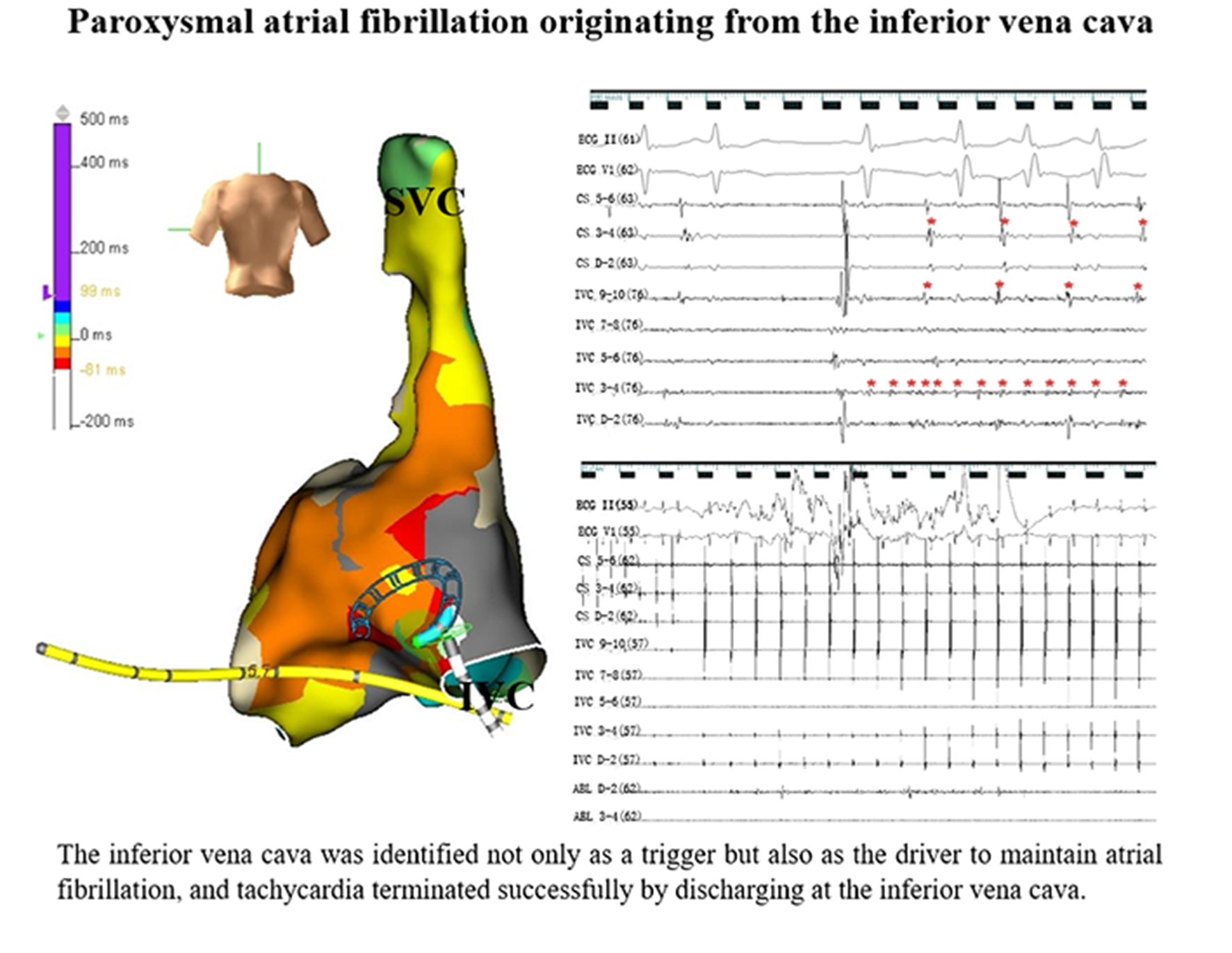

Supplement: Supplementary file 1 [file Image_1.TIF]
