# Supplementary material for: Paroxysmal Atrial Fibrillation Originating From the Inferior Vena Cava: A Case Report and Literature Review
Source: Front Cardiovasc Med. 2022 Jul 4;9:935524. doi: 10.3389/fcvm.2022.935524 (PMC9289394; doi:10.3389/fcvm.2022.935524)
Supplement: Supplementary file 2 [file Table_1.docx]

Supplementary Table 1. Diagnostic Assessment

| Diagnostic Assessment |  |
| --- | --- |
| Diagnosis | paroxysmal AF, atrial flutter, and atrial tachycardia |
| Diagnostic methods | ECG |
| Diagnostic challenge | prediction of AF origination based on surface ECG |
| Diagnostic reasoning | 1. ECG showed AF, atrial flutter, and atrial tachycardia 2. AF terminated spontaneously within 7 days of onset |
| Differential diagnosis | AF originating from extra-pulmonary vein |
| Prognostic characteristics | free of tachycardia after a 2-month follow-up |

Abbreviation: AF, atrial fibrillation; ECG, electrocardiogram; IVC, inferior vena cava; NA, not available; PVI, pulmonary vein isolation.
